# Postural Assessment Systems in the Prevention of Haemophilic Arthropathy: A Narrative Review

**DOI:** 10.3390/jfmk7030068

**Published:** 2022-09-07

**Authors:** Dalila Scaturro, Fabio Vitagliani, Sofia Tomasello, Maria Silvia Mangano, Gabriele Signa, Giulia Letizia Mauro

**Affiliations:** 1 Department of Surgical, Oncological and Oral Sciences, University of Palermo, 90127 Palermo, Italy; 2 Faculty of Medicine and Surgery, University of Catania, 95100 Catania, Italy; 3 Faculty of Medicine and Surgery, University of Palermo, 90127 Palermo, Italy

**Keywords:** haemophilia, balance, posture, musculoskeletal diseases

## Abstract

This narrative review aims to give an overview of some postural evaluation systems currently used in patients with haemophilia. Among them, first, we analyse the HJHS scale, recognized as a specific evaluation tool for haemophilic arthropathy. Second, we focus on other systems usually used in non-haemophilic patients that have also shown good applicability in this patient category, such as gait analysis, stabilometry, and baropodometric examination. This review underlines the use these tools could have in clinical practice to identify the early postural alterations in patients with haemophilia and set up personalised rehabilitation programs.

## 1. Introduction

Haemophilia is an X-linked inherited disease resulting from a factor VII (type A) or factor IX (type B) deficiency. On the basis of the serum levels of the corresponding deficiency factor, we can distinguish several forms: mild (<1%), moderate (1–5%), and severe (>5%) [1]. The most frequent and important complication that occurs in patients suffering from haemophilia is represented by repeated bleeding of the musculoskeletal system, especially inside the joints (haemarthrosis), which, in the long term, leads to the destruction of the articular cartilage and the consequent onset of chronic haemophilic arthropathy [2].

Hemarthrosis is very commonat the level of the synovial joints, in particular the knee and ankle, which are important for postural synergies [3]; if altered, they lead to alterations in postural balance and incorrect gaits, which often lead to the need for surgery [4,5,6,7]. This happens because repeated bleeding in haemophiliacs damages bone and ligament structures, altering information on joint mechanoreceptors, with a decrease in flexibility, strength, and proprioception [8,9]. For example, joint distension due to hemarthrosis can lead to the inhibition of the reflex of the extensor muscles and increase the activation of the flexors, which in turn leads to inaccurate information on the position and movement of the limbs [10,11,12,13]. The end result of this is impaired function and balance [9]. The imbalance could increase the risk of falls and kinesiophobia in this population, as well as favour a sedentary lifestyle, worsening musculoskeletal conditions and increasing the risk of bleeding and healthcare costs [14,15].

Posture can be considered an expression of the body’s static and dynamic adaptations to physical problems, even in the absence of clinical signs of arthropathy [16,17]. Given haemophilic boys’ greater tendency to develop postural disharmony, maintaining correct posture in this population could help prevent joint bleeding, chronic pain, and dysfunction [18]. This is why posture should be part of the routine evaluation in this population to allow the identification of dysfunction early and establish a tailored physical or rehabilitation program [18,19]. In this way, it will also be possible to better understand the joint conditions of the ankles and knees inpatients before surgery to more accurately recognise the effect of anexercise program on their balance [14]. The establishment of tailor-made exercise programs in haemophilia patients, which act on flexibility, strength, gait, and proprioception, helps to stop this negative cycle, protecting the joints from new bleeding episodes and improving patients’ quality of life [18]. Among these programs, proprioceptive rehabilitation plays an important role in promoting joint stability and function in all patients with haemophilia [9,20,21].

For the postural assessment of haemophilia patients, in addition to the specific Haemophilia Joint Health Score (HJHS) [22] questionnaire, the same postural assessment tools for healthy people are often taken into consideration, including Gait Analysis [23], stabilometric examination [24], and baropodometric examination [25].

We decided to conduct a narrative review of the literature to describe the main postural assessment systems used in haemophilia patients and to clarify their clinical applications.

## 2. Haemophilia Joint Health Score (HJHS)

The HJHS Score [22] is a tool that has been widely validated and is easy to use for the diagnosis and evaluation of haemophilic arthropathy over time. The HJHS measures the health of the joints most frequently affected by bleeding in patients suffering from haemophilia: the ankles, knees, and elbows.

It is designed to evaluate children (4–18 years) with haemophilia and mild joint impairment. It also may be used after orthopaedic surgery or as a measurement of outcomes during physiotherapy interventions.

Three paediatric studies have reported good interrater reliability with ICCs > 0.70. No data were found on Minimally Detectable Change (MDC) or Minimal Clinically Important Difference (MCID). Further research is needed to assess these data [26,27,28].

The HJHS was developed to be sensitive to small changes in joint health, particularly in countries where prophylactic treatment is used and when there is minor evidence of joint damage [26]. The HJHS was 97% more efficient than WFH in differentiating severe haemophilia from mild and moderate haemophilia and was 74% more efficient than the WFH in differentiating subjects treated with prophylaxis from those treated on request [29].

The current version, HJHS 2.1, consists of the evaluation of swelling (0–3); duration of swelling (0–1); muscular atrophy (0–2); crackling during movement (0–2); loss of flexion (0–3); loss of extension (0–3); joint pain (0–2) and strength (0–4) of the knees, ankles, and elbows; and an overall gait score (0–4). The maximum score is 124. A higher score indicates lower health of the articulation. Version 1.0 also included instability, gait for articulation, and axial alignment, with a maximum score of 148. Version 2.1 was adapted to evaluate the range of movement and gait, studying the patient globally rather than at the joint level [30].

The HJHS has been criticised by many authors because it is very expensive in terms of time for outpatient clinical practice [23,31]. Several studies have also demonstrated that both HJHS versions 1.1 and 2.1 are also valid tools for the assessment of arthropathy in adults [23,29,32].

According to den Uijl et al., the HJHS can be used to monitor joint bleeding in patients with moderate haemophilia to discover new complications and undertake extra treatment [32].

Fischer et al. reported that the HJHS could discriminate between different prophylactic strategies in people with different degrees of haemophilia and was responsive to changes following physiotherapy treatment [33]. By contrast, Nijdam A. et al. [34] pinpointed how HJHS assessment could be affected by observer bias, especially due to the different training and experience of the physiotherapists. These data suggest that the HJHS should be used mainly to monitor patients’ joint status over time or compare patients within centres or even per physiotherapist [33,34].

## 3. Gait Analysis

Gait analysis (or pace analysis) is particularly relevant in the assessment of haemophilia patients because it allows us to evaluate weight bearing in different joints. Gait analysis is a non invasive and very well-tolerated procedure. Therefore, it lends itself to be used in the paediatric populations as an early assessment tool, while in the adult, it allows the assessment of any deterioration of walking in patients with already established arthropathies [35,36].

It is performed by instrumentation consisting of a series of cameras (video or infrared) positioned around a walkway or a treadmill and connected to a computer. The patient has sensors located at various reference points on the body (e.g.,the iliac spines of the pelvis, the ankle malleolus, and the condyles of the knee) or groups of markers applied halfway up the body segments. Duringthe analysis, the computer calculates the trajectory of each marker in three dimensions. The vast majority of labs have also floor-mounted load transducers, also known as force platforms, that measure the reaction forces and the momentum of the reaction to the ground, including magnitude, direction, and position (called the pressure centre) [36]. Adding these data to the dynamics of each body segment allows the calculation of net forces and net force momentum on each articulation at each stage of the cycle of gait [37].

Three-dimensional gait analysis has been proposed as a valid and easily reproducible evaluation method [38] to monitor disease progression in patients with haemophilia. Children with haemophilia commonly show early functional deficits [39].

Axel Seuser et al. [40] observed significant differences between the study and control groups regarding the knee ROM, gait scores, and rhythmicity—changes in the angle of the knee during the stance phase—with almost no stretch position of the knee joint during heel strike; insufficient knee extension at toe-off or even a complete loss of stance phase activity; lack of regularity and rhythm in angular velocity and acceleration curves, which lead to minor efficiency of and greater fatigue; alterations in the axis of the legs during walking and knee flexion; acceleration peaks, especially in the stages of reversal of motion; and alteration of roll and glide movements. The authors addressed the physical examination as an important tool to detect subclinical symptoms of joint dysfunctions. Amotion analysis should then be taken into account to develop individualised physiotherapy, physical, or sport programs.

Stephensen D. et al. [41] highlighted significant alterations (*p* < 0.05) in kinematic and kinetics parameters of children with haemophilia. Increased bending angles and moments of strength outside the knee were also recorded, as well as higher external moments of plantar flexion of the ankle and external moments of hip flexion. These results, according to the authors, suggest that biomechanical changes already occur in the early stages of disease and that a certain degree of functional impairment may be present even ifthe clinical evaluation alone suggests otherwise.

Bladen M. et al. [42] evaluated the use of the GAITRite^®^ system (CIR Systems Inc., Clifton, NJ, USA) in haemophilia patients. It is made up of a 4.5m electronic walkway with built-in pressure sensors that record the position and relative pressure of each step. It provides an automatic calculation of 20 spatial and temporal parameters associated with gait and has been shown to provide reliable measurements. The authors pointed out that the GAITRite^®^ system is sensitive enough to highlight alterations of the gait in haemophilic boys who were still completely asymptomatic, pointing out significant increases in the timing of the oscillation phase and the single and double support phases.

Changes in spatiotemporal parameters were also highlighted by Forneris et al. [22] in patients with moderate or severe pathology. The changes in gait were also characterised by a frequent asymmetry of the load with an increase in the phase of support on the side opposite the target joint, a reduced phase of oscillation on the same side, a decrease in the average speed, and the presence of double asymmetric support (which was greater in subjects with motor difficulties). The authors identified one case of early haemophilic arthropathy using this approach, and other participants had therapy adjustments after the assessment. Therefore, according to Forneris et al., gait analysis is a useful tool to check the status of the joints in patients with haemophilia who are a symptomatic and allows clinicians to plan a personalised therapeutic–rehabilitative programme and monitor the clinical status over time.

## 4. Stabilometry

Stabilometry analysis involves the use of a system that includes a force place and an analogical–digital converter linked to a computer. Data collected are transferred into a computerised program that rearranges the data into a graphic showing the displacement of the Centre of Pressure (CoP), that is, the application point of the resulting force of all the vertical forces acting on the force plate; this reflects the balance ability: the movement the body performs to control the centre of gravity within the base of support (BOS) [43,44]. The musculoskeletal system fluctuates continuously to maintain balance; thus, postural control is necessary for rest and any physical activity. The authors said that “The interaction of the sensorial, nervous and musculoskeletal systems is responsible for the maintenance of postural stability” [43] and that “sensory inputs from joints and muscles are extremely important for the maintenance of postural orientation and equilibrium and the function of mechanical receptors can be impaired by repetitive joint or muscle bleeds” [44]. Thismeans that balance is reached by the coexistence of systems working together from the information obtained from the outside.

Several parameters canbe collected, such as the speed standard deviation and the width of CoP in the AP and LL projections in different orthostatic load positions: the eyes-open and eyes-closed bipodalic positions, eyes-open and eyes-closed on unstable surfaces (placing an irregular terrain between the feet and the force place), and monopodalic positions on the dominant leg and the non-dominant leg.

Fearn et al. [45], using the NeuroCom Balance Masteras a force place, measured the static postural sway speed (CoP mean displacement speed and sway degree); limits of stability in dynamics (reaction time: the time between the signal to move and the initiation of movement; maximum excursion (%):the furthest distance travelled by the centre of gravity away from upright stance); step and gait measurement (speed and width); and step quick turn, a measurement of the sway during a quick 180° turn on a single leg. According to their results, the static balance measurement showed a difference that was not statistically significant; however, the dynamic balance measurement showed worse performance in haemophilia patients.

Using a Kistler force place, Gallach et al. [43] analysed static balance by measuring CoP variation (CoP mean displacement speed, sway area, and mean frequency) in the PA and LM projections in haemophilia patients (with and without haemophilic arthropathy) compared with a control group in three different stances: an eyes-open bipodalic stance, an eyes-closed bipodalic stance, and a monopodalic stance with the contralateral leg at around 90°. Their results showed a statistically significant difference in bilateral stance trials for mean speed and sway width in both the PA and LM projections, with better performance in the control group. In addition, in the single-stance trials, the control group showed better results compared with haemophilia patients (worse results were obtained in patients with haemophilic arthropathy), with a statistically significant difference in mean speed and width.

Most studies have focused on adults; however, in haemophilic pre-adolescents, stabilometric analysis has also shown an increase in early instability and poor balance control compared with healthy patients, especially in situations that require more control [19,44,45].

F.M.B. Souza et al. [44] compared haemophilic children with healthy ones of the same age by using a force plate with the following sensorial conditions: eyes open (i) and closed (ii) on an even surface; eyes open (iii) and closed (iv) on an uneven surface. The authors measured the CoP sway and mean displacement speed and their ratios (visual VQ—ratio between (ii) and (i); proprioceptive PQ—ratio between (iii) and (i); vestibular VestQ—ratio between (iv) and (i)). The results showed a statistically significant difference in the PQ sway area value in whichthe haemophiliac group showed a higher quotient compared withthe control group. Balance evaluation is useful because, especially in haemophiliac pre-adolescents, who have a high demand for school physical activity, it allows for the establishment of a personalised training program, an adequate system of balance, and the prevention of bleeding.

Static disturbances of postural balance in the bipodalic standing position highlight the need for balance assessment in children with haemophilia. Increased mediolateral oscillations may be an early sign of musculoskeletal system disorders. The relationship between balance parameters and the clinical score of the lower limb joints suggests that static postural balance can improve by diagnosing balance disorders at an early age and by maintaining joint health through physiotherapeutic approaches [24].

## 5. Baropodometric Examination

Foot deformities are common in patients with haemophilic ankle arthropathy, including valgus/varus malalignment of the hindfoot and flat/hollow foot, due to distal tibial growth disorders, talar deformity, and subtalar involvement [46,47,48]. These deformities can become irreversible in the long term without appropriate treatment, and patients with foot deformities often experience discomfort while walking or standing for long periods.

In the immature joint, synovitis causes hypertrophy of the epiphyseal growth plates, and significant structural deficiencies can rapidly develop. This stimulus to the growth plates causes bone hypertrophy, discrepancies in leg length, and angular deformities. In mature articulation, haemophilia has an important harmful effect on articular cartilage. As it progressively worsens, the joint function deteriorates [24]. All these conditions predispose the patient to defects in gait and load distribution.

The baropodometric platform is thus also used in the clinical evaluation of joint instability in haemophiliacs. This system, integrated by a computerised mechanism, records the pressure exerted by the feet against the ground during the stance phase of the gait and allows the real-time display of these values during movement [49]. The weight and pressure of each foot are recorded individually in an upright position, and then a gait evaluation is performed. The result is a graphic that defines the trajectory of the pressure centre (CoP) at different times in the position from heel to toe. At the end of the test, there are as many lines as phases presented by the patient during the examination. From here, we can obtain the average peak/position, which is the average of all trajectories of the variation of the CoP.

In subjects with haemophilia, shortening of the load line is often reported, with an increase in load at the level of the midfoot, forefoot, and big toe, reducing it to the medial and lateral level of the heel. This generates backfoot instability and alterations in the correct gait and its phases.

Dysfunctions of the talocrural joint can alter the ROM in plantar flexion, dorsiflexion, or both. When the subtalar joint is not stable, medial or lateral deviations may occur with subversion or inversion of the back foot and subsequent repercussions at the level of balance posture and weight distribution; in subjects with haemophilia, this results in an increased risk of recurrent bleeding.

Joint instabilities, therefore, undoubtedly contribute to the onset of hemarthrosis in the ankles of haemophilia patients; computerised dynamic pedobarography is a reliable, reproducible, and risk-free means of assessment for the characterisation of the instability of the posterior foot and the ankles, allowing the possible prescription of an orthotic treatment to correct the instability and prevent subsequent bleeding of the backfoot and ankle [8].

## 6. Conclusions

The identification of alterations in posture and/or balance in children with haemophilia through the methods discussed plays a key role in allowing the implementation of corrective physiotherapy interventions based on proprioception, coordination, and stretching, with the aim of preventing the establishment of potentially harmful compensatory strategies. This allows patients with haemophilia to have a better quality of life and social life as well as greater functional independence. Finally, since it is a rare disease that requires a multidisciplinary approach, patients should be cared for and followed up in haemophilia-specific treatment centres so that they can receive full treatment.

## Data Availability

Not applicable.
